# GastricAITool: A Clinical Decision Support Tool for the Diagnosis and Prognosis of Gastric Cancer

**DOI:** 10.3390/biomedicines12092162

**Published:** 2024-09-23

**Authors:** Rocío Aznar-Gimeno, María Asunción García-González, Rubén Muñoz-Sierra, Patricia Carrera-Lasfuentes, María de la Vega Rodrigálvarez-Chamarro, Carlos González-Muñoz, Enrique Meléndez-Estrada, Ángel Lanas, Rafael del Hoyo-Alonso

**Affiliations:** 1 Department of Big Data and Cognitive Systems, Instituto Tecnológico de Aragón, ITA, María de Luna 7-8, 50018 Zaragoza, Spain; 2 Instituto de Investigación Sanitaria Aragón (IIS Aragón), 50009 Zaragoza, Spain; 3 Instituto Aragonés de Ciencias de la Salud (IACS), 50009 Zaragoza, Spain; 4 Centro de Investigación Biomédica en Red de Enfermedades Hepáticas y Digestivas (CIBERehd), 28029 Madrid, Spain; 5 Facultad de Ciencias de la Salud, Universidad San Jorge, 50830 Zaragoza, Spain; 6 Department of Gastroenterology, Hospital Clínico Universitario Lozano Blesa, 50009 Zaragoza, Spain; 7 School of Medicine, University of Zaragoza, 50009 Zaragoza, Spain

**Keywords:** artificial intelligence, gastric cancer, diagnosis, prognosis, clinical decision support tool

## Abstract

Background/Objective: Gastric cancer (GC) is a complex disease representing a significant global health concern. Advanced tools for the early diagnosis and prediction of adverse outcomes are crucial. In this context, artificial intelligence (AI) plays a fundamental role. The aim of this work was to develop a diagnostic and prognostic tool for GC, providing support to clinicians in critical decision-making and enabling personalised strategies. Methods: Different machine learning and deep learning techniques were explored to build diagnostic and prognostic models, ensuring model interpretability and transparency through explainable AI methods. These models were developed and cross-validated using data from 590 Spanish Caucasian patients with primary GC and 633 cancer-free individuals. Up to 261 variables were analysed, including demographic, environmental, clinical, tumoral, and genetic data. Variables such as *Helicobacter pylori* infection, tobacco use, family history of GC, TNM staging, metastasis, tumour location, treatment received, gender, age, and genetic factors (single nucleotide polymorphisms) were selected as inputs due to their association with the risk and progression of the disease. Results: The XGBoost algorithm (version 1.7.4) achieved the best performance for diagnosis, with an AUC value of 0.68 using 5-fold cross-validation. As for prognosis, the Random Survival Forest algorithm achieved a C-index of 0.77. Of interest, the incorporation of genetic data into the clinical–demographics models significantly increased discriminatory ability in both diagnostic and prognostic models. Conclusions: This article presents GastricAITool, a simple and intuitive decision support tool for the diagnosis and prognosis of GC.

## 1. Introduction

In recent years, technological advancements and digitisation have led to exponential growth in the generation of information. As a result of this widespread availability of data, there has been a growing demand for techniques capable of handling and analysing large amounts of heterogeneous information. Artificial intelligence (AI) has assumed a highly significant role in medicine, confronting medical challenges and demonstrating significant progress with remarkable outcomes. Specifically, AI techniques such as machine learning (ML) and deep learning (DL) have been studied and applied in various areas of medicine, addressing multiple objectives including predictive analysis, decision-making support, drug development, and treatment response, among others [1].

In the field of oncology, the application of AI has also been pivotal for early diagnosis, risk assessment, cancer prognosis, and treatment selection in a wide variety of cancers. In this context, numerous tools and systems have been developed to support clinicians in decision-making for a large number of cancer sites [2,3,4,5,6,7]. However, studies regarding gastric cancer (GC) tools and systems are very limited in the literature. Gastric cancer is a complex pathology which represents a serious global health burden. Although its incidence has decreased in most industrialised countries, GC still ranks as the fifth most common cancer and the third leading cause of cancer deaths worldwide [8]. It is well known that GC is a multifactorial disease resulting from complex interactions between environmental factors, lifestyle factors (namely dietary and smoking habits), *Helicobacter pylori* (*H. pylori*) infection, and host genetic factors [9,10,11]. However, the contribution of each factor to the risk and prognosis of GC is an area that still needs to be explored. In particular, remarkable advances in the analysis and understanding of the human genome have stimulated research on the role of genetic susceptibility in cancer development. To date, genome-wide association studies (GWASs) and meta-analysis have revealed a series of single-nucleotide polymorphisms (SNPs) involved in GC susceptibility and prognosis [12,13,14,15,16]. The low penetrance of most identified genetic variants means that they do not provide clinically relevant information on their own, but the combination of risk-associated alleles in a polygenic risk score (PRS) has been reported to represent a valuable method to identify subjects at risk of developing GC or presents an adverse outcome of the disease [17,18].

Most of the GC diagnosis tools reported in the literature are mainly based on endoscopic, histopathological, and computed tomography (CT) data, while prognosis tools focus on recurrence, metastasis, and survival prediction. Feng et al. [19] developed and validated a clinical decision support system based on radiomic features from CT to predict lymph node metastasis in GC using ML techniques. In addition, Hao et al. [20] designed a DL framework for predicting the survival of GC patients (SurvivalCNN) using both CT imaging data and non-imaging clinical data. However, these methods can be invasive and expensive, highlighting the need for more simple and cost-effective systems. In this context, Charvat et al. [21] developed a risk prediction model for GC that combines clinical and demographic characteristics, lifestyle factors, and biological markers, providing a simple scoring system to estimate the individual risk of developing the disease. A subsequent study by Mahmoodi et al. [22] reported a medical decision support system considering 27 effective features and evaluated the risk of GC using fuzzy cognitive maps (FCMs). Although this system demonstrated good accuracy and high agreement, the authors mentioned several limitations such as the use of a small sample size and the absence of external validation in clinical real practice. Like Mahmoodi’s study, many decision support tools and systems reported several barriers and limitations that should be considered. Most of these limitations are related to the lack of well-annotated and high-quality data for training, validating, and testing, small sample sizes, the use of standard statistical methods, and the lack of external validation, among others [23]. An additional hurdle related to AI-assisted diagnosis and prognosis tools is their insufficient usability and integration into clinical practice. This is largely due to the lack of explainability and interpretability of the models and results. In this context, Cabitza et al. [24] pointed out that the “black box” characteristic of algorithms may cause clinician’s suspicion of ML and lead to unintended negative consequences in clinical practice. Hopefully, advances in data visualization and interactivity tools have deepened the interpretation of algorithm decision-making, thus contributing to their optimization and widespread clinical acceptance.

Trying to address these issues, we developed a decision support tool (GastricAITool) for both the diagnosis and prognosis of GC, providing support to healthcare professionals in critical decision-making and enabling personalised strategies. The developed GastricAITool system is based on a multicentric dataset comprising 603 Spanish Caucasian GC patients and 643 healthy controls, including clinical–demographic, tumoral, environmental, and genetic information. In our study, various algorithms, both traditional and ML and DL, were explored and internally and externally validated. Additionally, explainable artificial intelligence (XAI) techniques were employed to provide transparency and interpretability of the final models. The outcome is an intuitive tool that allows clinicians to input patient data and obtain an assessment of GC risk and prognosis, accompanied by explanatory graphs that facilitate the clinician in analysing the reported result and understanding how the model reached that conclusion.

## 2. Materials and Methods

### 2.1. Study Population

The source of information based on which the study was developed was the results of a national case–control study conducted simultaneously in a network of 16 general hospitals integrated into the Spanish National Health System. A total of 603 unrelated Spanish Caucasian patients with primary gastric adenocarcinoma identified via endoscopic and pathological diagnosis and 643 cancer-free individuals with no history of gastrointestinal disease, matched by sex, age (±5 years), and area of residence were recruited at hospitals from May 2003 to August 2012. Patients with gastric neoplasms other than adenocarcinoma, secondary or recurrent GC, a previous history of other malignancies, and those that refused to participate were excluded.

At the time of inclusion, detailed information concerning date of diagnosis, age, gender, smoking habits, family history of GC, tumour location, histological subtype, and TNM staging (UICC/AJCC classification) was recorded. Gastric tumours were classified according to their histological type [25] as intestinal, diffuse, or indeterminate and by anatomical location as proximal and non-cardia or distal GC. Each participating hospital performed the follow-up periodically. Follow-up included computerised tomography of the chest and abdomen and haematological analysis at 3-month intervals during the first year, and thereafter at 6-month intervals. Moreover, an upper digestive endoscopy was performed every year. Information was updated by clinical specialists through in-person interviews, medical chart reviews, and, in some cases, direct calling. The latest follow-up data in this study were obtained in December 2023.

Most controls were blood donors and individuals recruited from the out-patient clinical services in the same hospitals as the cases. Individuals with evidence for past or present gastric ulcers, immunosuppressive disorders, and major systemic diseases such as lupus erythematosus, rheumatoid arthritis, or inflammatory bowel disease were excluded. Eligible controls were also interviewed with the same standard questionnaire designed for patients and information regarding demographic characteristics and potential risk factors including smoking habits and family history of GC was collected. All patients and controls gave written informed consent to the study protocol, which was approved by the Ethical Committee of the Hospitals. Following completion of the interview, 10 mL of peripheral blood from each participant (patients and controls) was collected for DNA extraction and the serological study of the *H. pylori* infection. Once processed, whole-blood and serum samples were aliquoted and stored at −80 °C until analysis.

### 2.2. Helicobacter Pylori Diagnosis

The *Helicobacter pylori* status in GC patients was assessed by means of both the urease test (CLO-test; Delta West Ltd., Canning Vale, Bentley, Australia) and the histological examination of biopsies taken at the antrum and corpus of the stomach during the endoscopic procedure. In addition, GC patients and controls were analysed to determine the presence of *H. pylori* infection and antibodies to CagA and/or to VacA antigens in the serum via Western blot analysis (Bioblot Helicobacter; Izasa, Barcelona, Spain). This test for *H. pylori* infection and CagA/VacA antibodies was previously validated in our area [26]. GC patients were considered positive for bacterial infection if any of the three tests was positive. A substantial agreement of the three methods was observed (kappa values > 0.65). However, for statistical and data analysis, only information related to Western blot analysis in serum samples from GC patients and controls was considered

### 2.3. Genetic Study: Selection of Polymorphisms and Genotyping

The panel of polymorphisms included in our study was selected a priori from the NCBI database “http://www.ncbi.nlm.nih.gov/snp (accessed on 6 August 2024)”, Genome build 38.p2., and the NHGRI-EBI GWAS Catalog “http://www.ebi.ac.uk/gwas (accessed on 6 August 2024)” based on three main criteria: (1) published evidence of an association with GC in GWAS or meta-analysis studies; (2) having reported a prevalence of at least 5% for the least frequent allele among Caucasians; or (3) having potential functional consequences leading to altered protein concentrations or protein functions. Finally, a total of 261 SNPs located in 116 genes related to gastric carcinogenesis were considered for analysis (Supplementary Material Table S1). In addition, a penta-allelic variable number of an 86 bp tandem repeat polymorphism (VNTR) in intron 2 of the *IL1RN* (interleukin-1 receptor antagonist) gene and 2 common deletion polymorphisms in the *GSTM1* (glutathione S-transferase) and *GSTT1* genes were included in this study. Genomic DNA was extracted from ethlyenedi-aminetetraacetic acid (EDTA)-preserved whole blood in an AutoGenFlex 3000 (Autogen Inc; Cultek, San Fernando de Henares, Madrid, Spain). Genotyping was performed at the Spanish National Genotyping Centre (CEGEN-Santiago de Compostela) using the Illumina Veracode Platform (Illumina, Eindhoven, The Netherlands), RFLP (restriction fragment length polymorphism)-PCR-based methods and TaqMan-MGB allelic discrimination assays. The VNTR polymorphism in intron 2 of the *IL1RN* gene and the *GSTM1* and *GSTT1* null genotypes were analysed by means of PCR according to previously described methods [27,28].

Among the 261 SNPs analysed, 18 were excluded from the final study due to failure of genotyping or an SNP call rate < 95% (rs1048771, rs11226, rs12917, rs1614984, rs1618536, rs1650697, rs174538, rs1760944, rs1801133, rs1805388, rs1863332, rs2228001, rs2228006, rs2228524, rs2308321, rs3212986, rs3737559, and rs861528). Samples in which more than 20% of the SNPs failed genotyping were excluded (cases = 13, HCs = 10). Finally, 243 SNPs and 3 *IL1RN*, *GSTM1*, and *GSTT1* gene polymorphisms in 1223 subjects (590 cases and 633 controls) were successfully genotyped and available for analysis.

### 2.4. Building GastricAITool

From the available database, the GastricAITool was developed, providing users with predictions for both GC diagnosis (GC risk probability) and prognosis (probability of death-free survival at different time intervals). The development process was analogous for both components diagnosis and prognosis, utilising specific techniques for each. This process involved several stages, ranging from data preprocessing and data analysis to feature selection, genetic risk score development, model selection, training, validation, and the exploration of interpretation and explanation techniques. Ultimately, the integration and creation of the clinical decision support system itself were carried out. The following sections detail the process and techniques used in each stage. Analyses were performed using R and Python language programming.

#### 2.4.1. Data Preprocessing

Given the extensive and varied nature of the dataset, the presence of missing values in the study variables was examined within the original dataset. The aim was to identify the most appropriate approach for data imputation and preparation to achieve comprehensive data. Concerning the genetic variables, 23 individuals possessing over 20% missing genetic information, as well as 18 SNPs with missing values exceeding 5%, were excluded from the dataset. These thresholds were set conservatively to ensure that the dataset retained high-quality and representative information. This approach maintained data integrity and reduces the risk of distorting the analysis due to the extensive imputation of potentially unreliable or artificial data. The remaining missing data were replaced with the most commonly occurring value within their respective grouping (either case or control). This methodology allowed for imputation consistent with the distribution of the data in the comparative groups, minimizing bias introduced by imputation and preserving the overall structure of the dataset.

After screening, the final dataset was divided into training and external validation sets at a ratio of 5:1. The training set was used in the model development, encompassing processes such as feature selection, algorithm choice, training, and selection model. The external validation set included the most recent 100 individuals diagnosed with primary GC and 100 cancer-free controls, matched by age, gender, and area of residence, and was used to provide a performance value of the final system. The selected 5:1 ratio prioritised a larger dataset for training, enhancing the model’s ability to learn from the feature space. The validation size was close to 20%, a commonly accepted size for evaluating the model’s performance on unseen data, ensuring a robust assessment.

#### 2.4.2. Data Analysis

An initial exploratory analysis was conducted to gain insights into the data, relationships between variables, and their impact on GC risk and survival. This analysis aimed to identify significant variables while minimizing noise and dimensionality. The significance level was set at 0.05 (*p* < 0.05). Genetic information was encoded considering additive, dominant, and recessive genetic inheritance models.

Regarding the diagnosis, variations in the distribution of variables between patient and control populations were evaluated. The chi-square test (χ^2^) was applied to qualitative variables, while the Mann–Whitney test was used to analyse the continuous variable of age. Furthermore, a univariate analysis was conducted, entailing the creation of logistic regression models, with each variable being treated as an input. Odds ratios (OR) and 95% confidence intervals (CI) were computed to assess the magnitude of the association between each variable and GC. For all tests, a two-sided *p*-value < 0.05 was considered statistically significant.

Concerning prognosis, the overall survival (OS) time in GC patients was calculated from the date of the diagnosis to the date of last contact or death from any cause. Patients who were still alive at the last contact and patients lost to follow-up were considered as censored events in the analysis. This analysis was carried out across four clinically significant time intervals: 1.5, 3, 5, and 10 years. Overall survival for each variable was estimated by Kaplan–Meier survival curves and compared using log-rank test [29] when the proportional hazards assumption was satisfied. In cases where it was not, the Wilcoxon (or Gehan-Breslow) test was used [30].

#### 2.4.3. Genetic Risk Score

With the aim of quantifying and summarizing the genetic risk associated with GC, genetic risk scores (GRSs) were developed by combining gene variants into a single measure. Starting from the significant SNPs identified in the univariate analysis, a stepwise multivariate logistic regression model was implemented for genetic variables in diagnosis, and a Cox proportional hazards regression model was employed for prognosis.

The selected genetic variables were utilised to compute the GRSs. The number of risk alleles was encoded as 0, 1, or 2 for each SNP, assuming a log-additive genetic effect. Two types of GRS (weighted and unweighted) were calculated. The unweighted GRSs assumed equal effects for all SNPs and were obtained by summing the risk alleles of the selected SNPs. Conversely, the weighted GRSs considered distinct effects for each SNP, obtained by summing the risk alleles multiplied by their respective coefficients from the regression models.

In order to study the association between GRS and GC risk, regression models were constructed, and the corresponding OR and 95% CI were calculated. Similarly, the association between GRS and OS time was analysed through hazard ratios (HR). A two-sided *p*-value < 0.05 was considered statistically significant.

#### 2.4.4. Data Modelling

Considering the selected variables and constructed GRS, regression models were developed using various input combinations (non-genetic, genetic, and their combination) for comparison. The primary aim was to pinpoint optimal input combinations that would yield superior predictive outcomes, assessing whether integrating new information significantly improves model performance. The combination of input variables that demonstrated the best performance were subsequently selected as the input variables for the final model.

To build the final diagnostic and prognostic models, different traditional, ML, and DL algorithms were explored. For the diagnostic model, the following algorithms were analysed: logistic regression (LR) (with and without regularization, Lasso and Ridge) [31], Random Forest (RF) [32], Support Vector Machine (SVM) [33], eXtreme Gradient Boosting (XGBoost) [34], and Multi-layer Perceptron (MLP) [35]. For the prognosis model, following algorithms, designed for survival analysis, were explored: Cox Proportional Hazards regression [36] (with and without regularization, Lasso, and Ridge), Random Survival Forest (RSF) [37], Survival Support Vector Machine (SSVM) [38], Survival XGBoost, and a DL-based model, the Cox proportional hazards deep neural network (DeepSurv, [39]). To select the optimal model, a hyperparameter space was examined for each ML algorithm. Hyperparameter optimisation was conducted using the Optuna framework [40], which enables a dynamic search space through the tree-structured Parzen estimator algorithm [41].

The discriminative capacity of the diagnostic models was assessed by calculating the area under the ROC curve (AUC), whereas the performance of the prognosis models was measured using the Harrell C-index. This evaluation was conducted through five-fold cross-validation on the training dataset. Moreover, the test for comparing ROC curves proposed by Pepe et al. [42] was applied to assess the difference between diagnostic models with different inputs. Additionally, the discriminative capacity of the model and its generalisation were evaluated on the external validation set, as well as its calibration. This evaluation took into account data that have not been previously used in the process.

#### 2.4.5. Interpretability and Explainability

Several XAI techniques for model interpretability and explainability were investigated, providing insights into the internal functioning of the ML model. These techniques are visually represented through graphics. Both global explanation techniques, which offer an understanding of the overall behaviour of the model, and local explanation techniques, which provide details on how the model makes predictions for individual cases, were explored [43]. The most suitable ones were selected and implemented in the final diagnostic and prognostic tool.

#### 2.4.6. Clinical Decision Support Tool: GastricAITool

Once the models and their interpretability techniques were constructed, they were integrated into the GastricAITool system, a tool designed to assist in clinical decision-making for the diagnosis and prognosis of GC. The GastricAITool tool was implemented within an OKD infrastructure [44], featuring a NodeJS [45] FrontEnd client and a RestAPI backend server supported by a PostgreSQL database [46]. The distinguishing feature of the integrated system is its modularity and flexibility, allowing for continuous adaptation and development. Specifically, the system consists of three components:

- Frontend User Interface: This is the visual component through which users interact with the GastricAITool tool. It allows users to input data, configure options, and visualise results. It was built using technologies like Vue.js [47], Nuxt.js [48], and Vuetify [49].
- GastricAITool Model Executor/Manager: This component is responsible for processing input data, applying the model, and generating corresponding results. It is programmed in Python.
- PostgreSQL Database: Responsible for storing and managing the data necessary for the tool’s operation, including user input data and the results generated by the model.

GastricAITool was integrated into the GateKeeper platform provided by the European project https://www.gatekeeper-project.eu/ (accessed on 6 August 2024). The ultimate goal of this system is to support collaboration and data sharing in the realm of healthcare and value-based care. In this regard, the partners (Technological Institute of Aragón and Instituto de Investigación Sanitaria Aragón) presented these services at the GateKeeper Marketplace https://gatekeeper-marketplace.iti.gr/vendor/ITAINNOVA-IISA/ (accessed on 18 September 2024). Thus, the GastricAITool (version 01.10.0) is offered as a free service within the Marketplace, allowing its use by both clinicians and researchers, as well as its future development, leveraging the modularity and flexibility of the integrated system.

## 3. Results

### 3.1. Data Analysis

Clinical and demographic characteristics of GC patients and controls in the training set are shown in Table 1. Among the evaluated features, active smoking (OR: 1.57; 95% CI: 1.15–2.17), infection with *H. pylori* (OR: 1.58; 95% CI: 1.22–2.06), and a positive family history of GC (OR: 2.61; 95% CI: 1.71–4.06) were significantly associated with GC risk in the univariate analysis. Age and sex did not exhibit significant differences between GC patients and controls due to the construction of the database, where cases and controls were matched by sex and age.

Based on these findings, active smoking, *H. pylori* infection, and a positive family history of GC were chosen as input variables for the construction of the diagnostic model. Additionally, age and sex were also included based on clinical criteria. Regarding genetic variables, out of the 246 polymorphisms initially evaluated, 15 SNPs and a VNTR polymorphism in intron 2 of the *IL1RN* gene were significantly associated with GC risk (Table 2) and, therefore, were selected for the construction of the diagnostic model.

**Figure 1:**
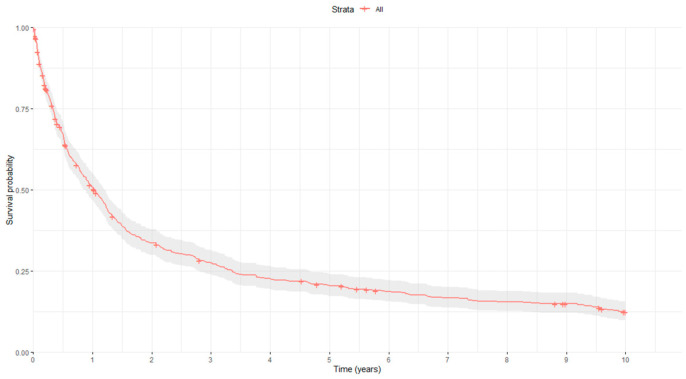
Kaplan–Meier survival curve. Red curve represents the estimated survival, while the gray area surrounding it indicates the confidence interval of the Kaplan-Meier estimator.

Concerning prognosis, the median follow-up time for all GC patients in our study was 11.21 months (range: 0.03–120). Four hundred and eighty-five GC patients (82.2%) had died at the end of the follow-up period (May 2003–December 2023), with the median OS being 12.33 months (10.59–14.60). Figure 1 shows the overall survival curve of the study population.

**Figure 2:**
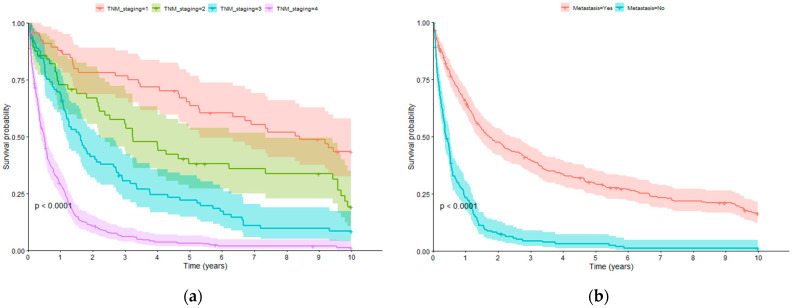
Overall survival curves: (**a**) TNM staging; (**b**) metastasis at diagnosis time.

Table 3 shows the *p*-values after the comparison of OS curves at different time instants (1.5, 3, 5 and 10 years) according to demographic, clinical, and tumour-related characteristics of GC patients. In the univariate analysis, proximal location of the tumour (HR: 1.549; 95% CI: 1.208–1.986), and advanced tumour stages (III and IV) (HR: 3.249; 95% CI: 2.505–4.213) were associated with significantly reduced OS, whereas surgical treatment (HR: 0.315; 95% CI: 0.253–0.393), chemotherapy (HR: 0.757; 95% CI: 0.615–0.932), and radiotherapy (HR: 0.590; 95% CI: 0.428–0.812) were related to a better prognosis of the disease. Based on these findings, the following characteristics were selected as input variables for the construction of the prognostic model: (a) clinical–demographic variables: sex (although not significant in any of the four ranges, it is a variable of clinical interest) and age; (b) tumour-related variables: tumour location, TNM staging, and presence of metastasis; and (c) treatment-related variables: whether chemotherapy, radiotherapy, and surgery have been received (regardless of whether it was a single or combined treatment). Specifically, the variables related to TNM information and the presence of metastasis at the moment of the diagnosis had a notable impact on OS time among GC patients (Figure 2).

Concerning genetic information, 26 genetic variants were significantly associated with OS in the univariate analysis (Table 4) and selected for the construction of the prognostic model.

### 3.2. Genetic Risk Scores

Weighted and unweighted GRSs were evaluated in our study. The constructed unweighted GRS reflects the sum of risk alleles. However, representativeness is not always guaranteed, particularly at the extremes. Due to this representation imbalance, categories were grouped at the extremes as follows. Concerning weighted GRS, GRS quartiles were considered in order to facilitate the analysis of the association with GC risk.

**Figure 3:**
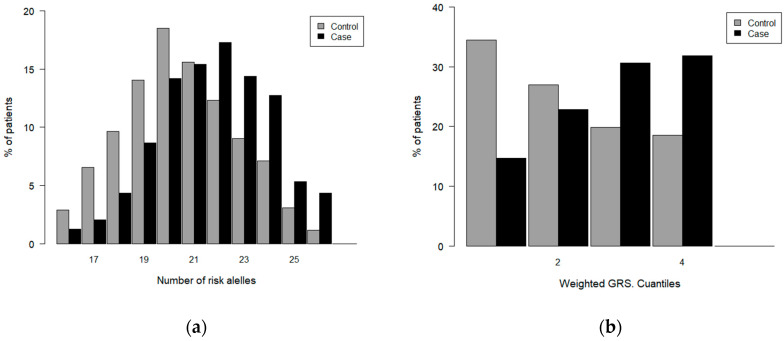
Distribution of GRS values in case and controls. Diagnosis: (**a**) unweighted GRS; (**b**) weighted GRS.

Figure 3 displays the distribution of the GRS values (unweighted and weighted) in GC patients and controls, constructed for the diagnostic model. Both populations follow a normal distribution with a maximum value of 20 risk alleles for the control group and 22 for the GC patient group, the value at which a shift occurs toward a higher percentage of high GRS values in patients compared to the controls (Figure 3a). A similar trend was observed for the distribution of weighted GRSs in patients and controls (Figure 3b).

The association between the unweighted GRS and GC risk is shown in Table 5, where the lowest value was considered as the reference value. Significant associations between high GRS values and the risk of GC were found. Specifically, individuals with 26 risk alleles showed a significant increase (12.25 folds) in the risk of GC compared to subjects with a GRS ≤ 15. The GRS considered as a continuous variable demonstrated a significant association with a 25% increased probability of GC per unit increase.

The weighted GRS exhibits a similar behaviour, as depicted in Table 6, considering quartile grouping.

Similar conclusions were reached for prognosis, where the analysis revealed a significant association between the GRS value and OS time, suggesting that higher GRS values are associated with a more unfavourable prognosis (Table 7 and Table 8).

### 3.3. Data Modelling

Regarding diagnosis, eight different models were developed using logistic regression analysis. These models range from a clinical–demographic model to univariate models that use the GRS as an input, as well as multivariate models combining both genetic and non-genetic information. The results obtained by these models are presented in Table 9.

The analyses revealed that the weighted GRS achieved slightly better models than the unweighted GRS. Moreover, applying the curve comparison test, we found that incorporating genetic information into the clinical–demographic model significantly enhances the model’s accuracy (from AUC = 0.606 to 0.682), and vice versa (from AUC = 0.647 to 0.682). Nevertheless, for simplicity and based on the achieved AUC value, the combination of input variables from model 5 was chosen: age, sex, smoking, *H. pylori* infection, family history of GC, and weighted GRS.

Regarding prognosis, comparisons were conducted among seven different models using Cox regression. Table 10 displays the performance achieved by these models.

As in the diagnosis, similar conclusions were obtained after comparing prognosis models. Thus, incorporating genetic information into the non-genetic model increased discriminatory ability (C-index from 0.664 to 0.761), and vice versa (C-index from 0.73 to 0.761). In our study, the best model was achieved by considering all non-genetic variables and the weighted GRS.

Considering the selected variables as the input, Supplementary Material Tables S2 and S3 display the explored hyperparameter search range, as well as the optimal configuration achieved for each investigated diagnostic and prognostic algorithm, respectively. The discriminative capacity of each algorithm considering its optimal configuration is shown in Table 11.

Despite other algorithms being close, the XGBoost model achieved the most remarkable performance (AUC = 0.684) for diagnosis. This model was chosen as the definitive diagnostic model. On the other hand, the DL model, DeepCox, achieved the best performance in prognosis. However, for the sake of interpretability, the Random Survival Forest model was chosen as the final model, as it closely matches DeepCox’s performance and allows for the application of explanatory techniques.

The final diagnostic model achieved an AUC of 0.631 in the external validation set. The optimal cutoff point was around 0.5, as expected due to the class balance resulting from the construction of the database (case–control). As for the prognostic model, it achieved a C-index of 0.7143 in the external validation set, with similar values when considering models with censoring at 1.5, 3, and 5 years. In terms of calibration, in the diagnostic case, an intercept of 0.17 and a slope of 0.8 were estimated in the external validation set. For the prognosis, the integrated time-dependent Brier score was used, providing an overall performance calculation across all available time points, resulting in a value of 0.15. All of these values suggest a reasonable calibration of the model.

#### 3.3.1. Interpretability and Explainability

**Figure 4:**
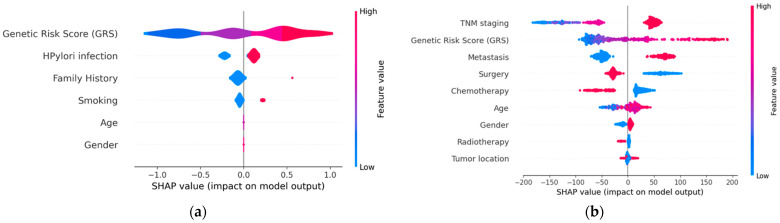
Global explainability: (**a**) diagnosis; (**b**) prognosis. (**a**) indicates that genetics, *H. pylori* infection, family history of GC, and smoking are the factors that globally influenced the model’s decision the most in this example. High GRS values and having a family history of GC are associated with an increased risk of GC. Conversely, low GRS values or not being infected by *H. pylori* act as protective factors. (**b**) indicates that TNM stage and genetics are the factors that most significantly influenced the model’s decision globally, while tumour location had the least influence. Advanced TNM stages, high values of the GRS, having metastasis at the time of diagnosis, not undergoing surgery, and not receiving chemotherapy are factors with a negative impact on survival (less favourable prognosis).

The selection of graphical representations that enable the understanding of the models and the results they provide, resulting from the implementation of XAI techniques, was carried out considering clinical criteria. Both global explainability graphics, providing an overall understanding of the model, and local explainability graphics, focusing on explaining how the model makes specific decisions for individual instances, were chosen. The chosen graphs were constructed based on the Shapley technique. Figure 4 displays the global explainability plots for the final models. The position on the *x*-axis is determined by the Shapley value, while on the *y*-axis, the variables are located, ordered from highest to lowest importance. The further away from the vertical line, the more pronounced the variable’s contribution. Positive Shapley values indicate a risk of GC, whereas negative values indicate the opposite. The colour reflects the value of the variable, using blue for lower values and red for higher values.

**Figure 5:**
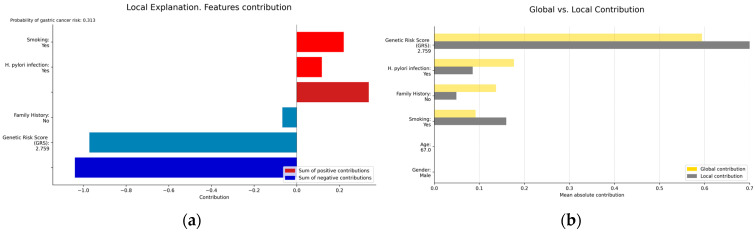
(**a**) Local explainability; (**b**) local vs. global explainability. (**a**) illustrates the local explainability graph of an individual who, despite been infected by *H. pylori* and being a smoker, is at low risk of GC due to lacking a family history of the disease and having a low GRS (2.7 out of 10). Additionally, (**b**) represents both the local and global explainability of the model. The mean absolute contribution of each variable to the model’s prediction is presented. This graph allows for the comparison of variable importance on a global level and in individual predictions. In this example, the GRS presents a more significant contribution compared to its contribution in the global model, acting as a protective factor.

**Figure 6:**
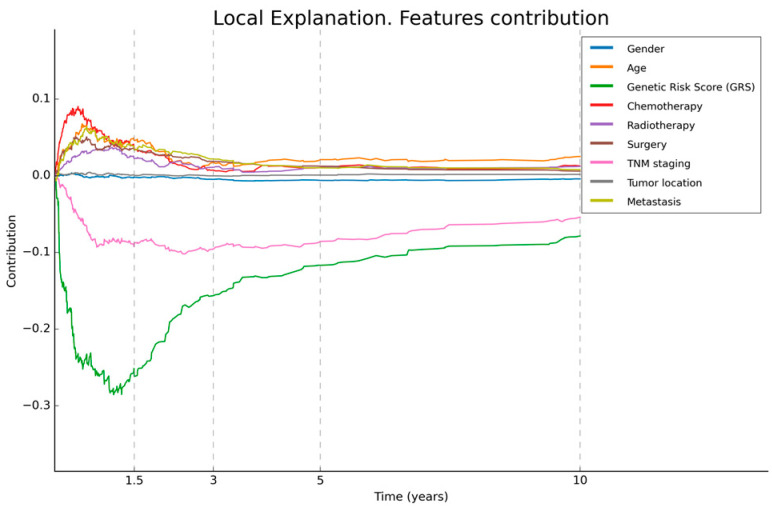
Local explainability of prognosis. This figure illustrates the local explainability graph for a 40-year-old male patient with distal GC and an advanced TNM stage IV. In addition, this individual underwent chemotherapy, radiotherapy, and surgery after his diagnosis. His GRS value is 10 (maximum value). The graph depicts the contribution of each variable over time. Negative contributions indicate a higher risk (less favourable prognosis), while positive contributions indicate the opposite. The graph shows that, despite the patient presenting only two characteristics that have a negative influence on overall survival, these factors have a significant impact, reaching the highest levels.

In order to visualise the local explainability, several graphs representing the contribution of each variable in the model’s decision for a specific patient were generated for both the diagnosis (Figure 5) and prognosis (Figure 6) models.

#### 3.3.2. Clinical Decision Support Tool: GastricAITool

**Figure 7:**
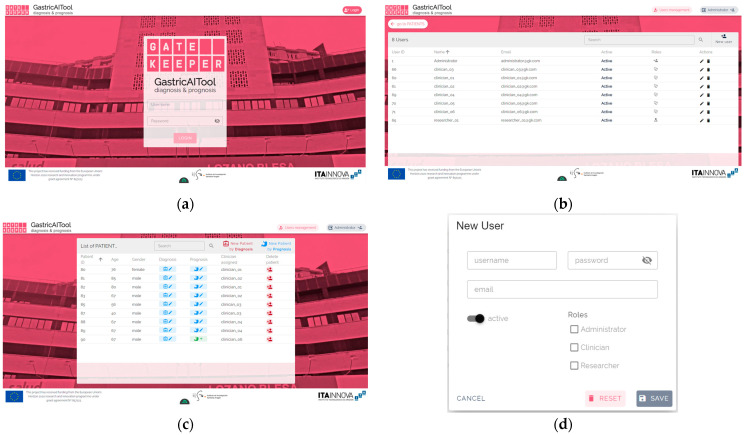

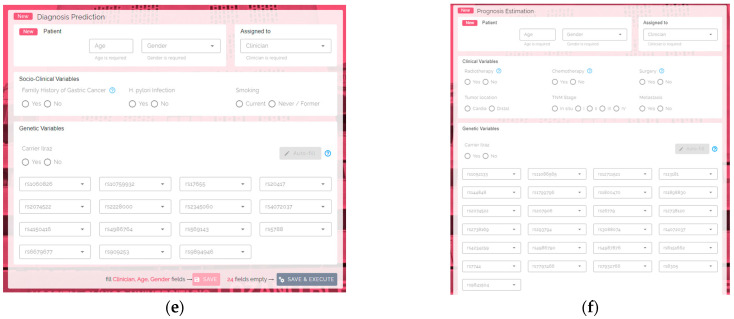
GastricAITool interface. (**a**) Main screen. (**b**) list of users of the platform. (**c**) list of patients. (**d**) creation of a new user. (**e**) diagnosis variables input; (**f**) prognosis variables input. (**a**) displays the main screen of the tool, where the user must login by providing their username and password. (**b**) User management system screen. (**c**) Three roles are distinguished: administrator, clinician, and researcher. Only the administrator is authorised to manage users, the clinician has the remaining permissions, while the researcher has read-only permissions. (**d**) The first screen that appears once logged in is the patient list along with options to edit data, create, or delete patients. The patient’s age and gender information are displayed, along with their assigned clinician. (**e**,**f**) show the screens that appear when adding a new patient to predict their diagnosis (**e**) or prognosis (**f**). These screens display the necessary patient information that needs to be completed. Additionally, an option is provided to autofill genetic information if it is unavailable, as long as at least 80% of the required genetic variables have been filled. Once all the information has been filled out, the tool allows saving the data and/or executing the diagnosis or prognosis model.

**Figure 8:**
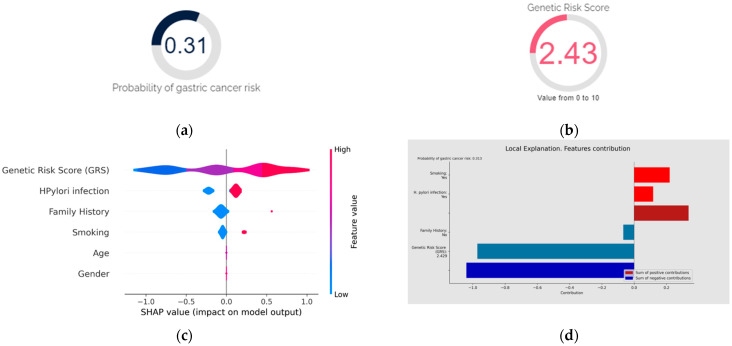

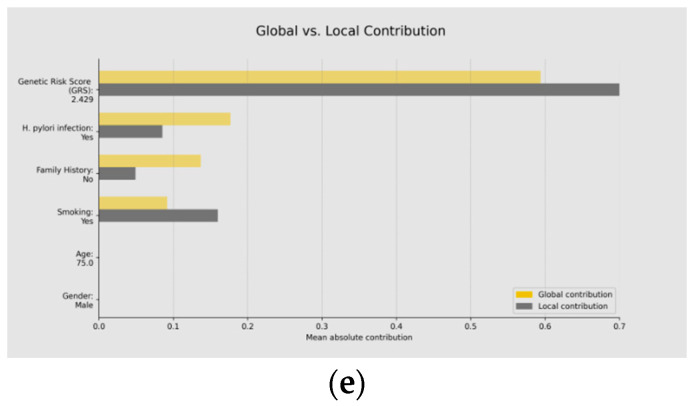
Diagnosis using GastricAITool. (**a**) Probability of GC risk of the individual; (**b**) GRS of the individual; (**c**) variables’ global explanation in GC risk; (**d**) local explanation in GC risk; (**e**) global vs. local explanation in GC risk. For the diagnosis scenario, the tool provides the probability of GC risk, the model’s results, the GRS value (**b**), and the global (**c**) and local (**d**) explainability graphs, along with the corresponding help functions. In the example shown in (**c**), genetics, *H. pylori* infection, family history of GC, and smoking are the factors that globally influenced the model’s decision the most. High GRS values and having a family history of GC are associated with an increased risk of GC. Conversely, low GRS values or not being infected by *H. pylori* act as protective factors. (**d**) illustrates the local explainability graph of an individual who, despite been infected by *H. pylori* and being smoker, is at low risk of GC due to lacking a family history of the disease and having a low GRS (2.4 out of 10). Positive contributions are highlighted in red, indicating GC risk, while negative contributions represent the opposite. In this example, the sum of negative (protective) contributions is greater than the positive (risk) ones. Additionally, (**e**) represents both the local and global explainability of the model. The mean absolute contribution of each variable to the model’s prediction is presented. This graph allows for the comparison of variable importance on a global level and individual predictions.

**Figure 9:**
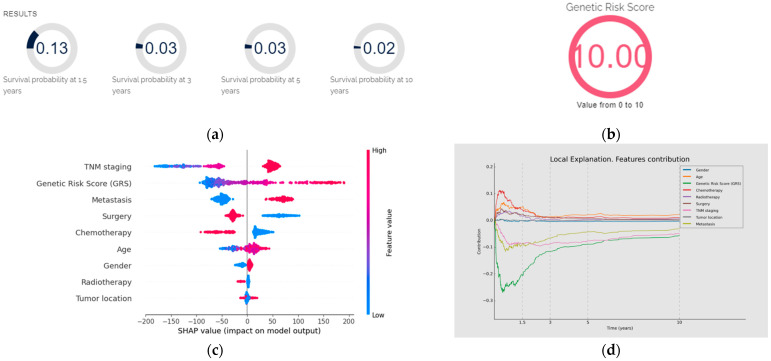

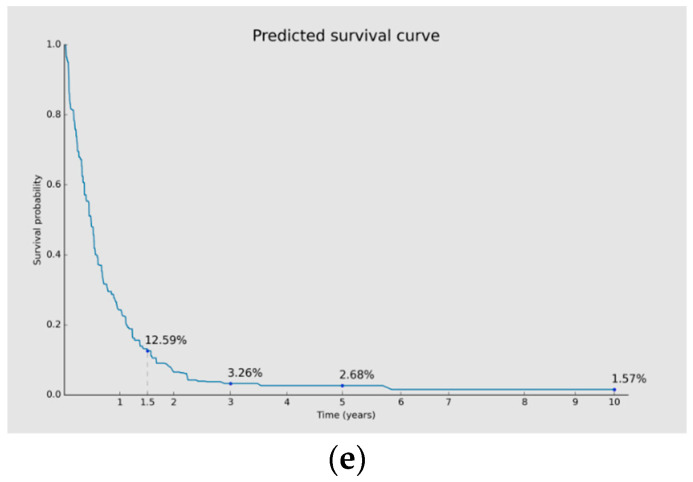
Prognosis using GastricAITool. (**a**) Probability of survival of the patient; (**b**) GRS of the patient; (**c**) variables’ global explanation in GC prognosis; (**d**) local explanation in GC prognosis; (**e**) survival curve and probabilities of survival. (**a**,**b**) illustrate the probability of survival of the patient (at 1.5, 3, 5 and 10 years) and the value of the GRS, respectively. (**c**) indicates that, in this patient, TNM stage and genetics were the factors that most significantly influenced the model’s decision globally, while tumour location had the least influence. Advanced TNM stages, high values of the GRS, having metastasis at the time of diagnosis, not undergoing surgery, and not receiving chemotherapy were factors with a negative impact on overall survival. (**d**) illustrates the local explainability graph for a 40-year-old male patient with distal GC and an advanced TNM stage IV. In addition, this individual underwent chemotherapy, radiotherapy and surgery after his diagnosis. His GRS value is 10 (maximum value). The graph depicts the contribution of each variable over time. Negative contributions indicate a higher risk (less favourable prognosis), while positive contributions indicate the opposite. The graph shows that, despite the patient presenting only two characteristics (GRS and TNM) that have a negative influence on overall survival, these factors have a significant impact. By contrast, being 40 years old and having received chemotherapy positively affect the prognosis, especially during the first months of the follow-up. (**e**) displays the probabilities of survival (without mortality) at 1.5, 3, 5, and 10 years. Additionally, the predicted survival curve is presented.

The ultimate goal of this project was to develop and implement a diagnostic and prognostic tool for GC called GastricAITool. GastricAITool provides functionalities including user management, the creation and editing of patient data, and the visualization of diagnostic and prognostic model results (Figure 7). For the diagnosis scenario (Figure 8), this tool provides the probability of GC risk (results of the model, GRS values, global and local explainability graphs, and the corresponding help functions). Regarding prognosis (Figure 9), the probabilities of survival at 1.5, 3, 5, and 10 years are displayed along with the predicted survival curves.

## 4. Discussion

GC is a complex condition that represents a significant burden on global health [50]. It is now widely accepted that GC arises from the interaction of diverse elements, ranging from clinical and demographic factors to bacterial and genetic factors. Therefore, it is crucial to delve deeply into how these factors influence the risk and prognosis of GC, while concurrently developing decision support tools that facilitate accurate diagnosis and the creation of personalised strategies. The outbreak of the COVID-19 pandemic emphasised the relevance of quickly and effectively addressing healthcare systems by accurately diagnosing at-risk patients and prioritising medical attention. In this context, AI has assumed a pivotal role, driven by technological advancements and digitalisation. However, despite the benefits offered by techniques like ML and DL, challenges persist that need to be addressed. Furthermore, while several cancer decision support tools have been developed [2,4,5,6,7], those specifically focused on GC remain scarce.

In response to this demand, we created GastricAITool, a clinical decision support tool for the diagnosis and prognosis of GC. GastricAITool is based on a multicentric Spanish study comprising 603 Caucasian patients with primary GC and 643 healthy controls. The resulting database includes a wide variety of information concerning age, gender, lifestyle habits, family history of GC, comorbidities, tumour location, histological subtype, TNM staging, therapy (chemotherapy, radiotherapy, and surgery), and GC risk- and prognosis-related gene polymorphisms. Most of GC decision support tools reported in the literature are based on imaging and basic demographic data [19,20,21,22]. However, very few studies account for other relevant factors such as modifiable lifestyle factors and environmental and genetic factors [51,52]. In the era of personalised medicine, robust clinical decision support tools for the early diagnosis and prediction of adverse outcomes require the integration of all this information in order to achieve a more effective approach to patient care.

In our study and after performing the initial data preprocessing, an exploratory data analysis aiming to determine the influence of clinical–demographic variables on CG risk was conducted. The most relevant features were then selected, thus reducing the problem’s complexity. In agreement with previous studies [9,53,54], our results showed that tobacco smoking, *H. pylori* infection, and a family history of GC were significantly associated with GC risk. Although it is well documented that GC is more common in males and the elderly [55], we found no significant differences regarding sex and age between GC patients and controls in our population. This is due to the methodology employed in constructing the original database, which involved sex and age matching between cases and controls. Consequently, the relevance of these two features as variables in the risk prediction models could not be assessed. Concerning prognosis, TNM-related variables, the presence of metastasis at the moment of the diagnosis, and the receipt of chemotherapy, radiotherapy, and/or surgery had a notable impact on overall survival in our cohort of GC patients. In line with our findings, TNM stage and treatment-related variables have been described as common prognostic factors in the literature [56,57]. Gastric cancer is one of the most lethal malignant tumours, with a five-year survival rate of <30% in Western countries [58]. In our study, most GC patients presented with late-stage disease and showed an overall survival of less than 3 years, highlighting the unfavourable prognosis of this disease and the need for more effective prevention measures and follow-up surveillance.

In relation to the genetic study, 16 polymorphisms out of the 264 initially evaluated were ultimately selected for the construction of the diagnostic models, and 26 were selected for the construction of the prognostic models. Of note, only the rs2074522, rs4072037, and IL1RN*2 variants were chosen for both the diagnostic and prognostic models, emphasizing the relevance of performing separate analyses for each, as carried out in this study. Xin et al. [52] yielded similar conclusions after evaluating the association of GWAS-reported risk variants across 17 cancer types and overall survival in two large-scale European cohorts. The authors found no associations between cancer risk-related variants and overall survival in patients, supporting the different role of risk variants in cancer survival prediction. Recently, Duan et al. [18] reported a GC risk prediction model based on non-genetic (age, gender, smoking status, drinking status and *H. pylori* infection) and genetic factors, including 21 lncRNAs and 20 common SNPs significantly related to GC in the Chinese population. Interestingly, 12 of the 20 SNPs included in Duan’s model were also evaluated in our study. However, only the *MUC1* rs4072037 and *PTGS2* rs20417 variants were associated with GC risk in our study. Likewise, Ni et al. [59] identified a panel of 26 SNPs significantly associated with OS in two cohorts of Chinese GC patients. However, our study failed to replicate any associations. Besides differences regarding methodology and sample sizes, a potential explanation for these discrepant results is the large variation in allele frequencies observed among populations. As a result, the generalizability of predictive models to other populations beyond the population studied may represent a major limitation in some studies. Therefore, well-designed studies with large sample sizes targeting different geographic areas and ethnic groups should be conducted to ensure a more effective generalization. 

Most genetic variants identified in our study do not provide clinically relevant risk by themselves, but the combination of risk alleles in a polygenic model has been reported to increase the risk in an additive or exponential way. Genetic risk scores provide an easily single interpretable value representing the sum of risk alleles. In fact, the elaboration of GRS is becoming a very attractive issue showing very promising results to identify subjects at risk of developing cancer or presenting an adverse outcome of the disease [60,61,62]. In our study, the constructed GRS exhibited a significant influence on the diagnosis and prognosis of GC. In particular, we found that the risk of developing GC increased along with the number of risk alleles, with an almost 12.3-fold increased risk in carriers of ≥26 risk alleles compared with subjects with a GRS of ≤15. Similar conclusions were reached for prognosis, where the analysis revealed a significant association between higher GRS values and lower OS time. In addition, GRSs have been reported to substantially improve the accuracy of diagnostic and prognostic assessments when integrating with phenotypic variables [63,64,65]. In line with these findings, our study showed that incorporating GRSs into models using clinical and demographic data alone significantly increased discriminatory ability in both, diagnostic (from AUC = 0.606 to 0.682) and prognostic (C-index from 0.664 to 0.761) models. Our results suggest that the addition of a GRS to clinical models has great potential in the prediction and prognosis of GC and would benefit the development of individual risk scores and more effective screening strategies.

Unlike other studies, our work addressed a variety of analysis methods ranging from traditional approaches such as logistic and Cox regression to widely used ML and DL algorithms. Each evaluated algorithm has its own specific characteristics, advantages, and limitations. Logistic regression and Cox regression are effective algorithms for modelling linear relationships and are inherently interpretable. However, their main limitation is their difficulty in effectively addressing complex and nonlinear problems [31,36]. In contrast, SVM can handle nonlinear relationships using kernels, offering greater flexibility in data modelling, although they lack inherent interpretability [33]. Ensemble algorithms like Random Forest and XGBoost combine multiple trees to capture interactions between variables and improve overall performance, while also reducing the risk of overfitting [32]. Unlike Random Forest, which builds trees independently, XGBoost builds trees sequentially which can have a significant impact on model performance. Generally, XGBoost provides better results in practice, often outperforming other machine learning and conventional approaches in various applications [34]. The global interpretation of these models can be challenging due to their complexity and the large number of trees. However, current XAI techniques can address these limitations [43]. Finally, the MLP is effective at modelling complex nonlinear relationships and has shown good performance in problems with intricate patterns. However, this model requires large volumes of data for effective training [35].

While state-of-the-art results can provide some useful insights into algorithm performance, the effectiveness and selection of the best algorithm depend on the specific data and problem at hand. In agreement with previous works [66,67], the XGBoost algorithm achieved the best performance in predicting GC risk in both the training (AUC = 0.684) and the external validation (AUC = 0.631) sets. AUC values in our study were slightly lower than those reported by other studies considering lifestyle factors and subjects’ non-invasive characteristics [68,69,70]. The model for diagnosing GC in our study was based on relevant risk factors for GC development such as *H. pylori* infection, tobacco smoking, family history of GC, and gene polymorphisms. However, other well-known risk factors such as sex and age were not taken into account due to the matched design of the dataset. Therefore, there is potentially significant room for improvement by properly considering sex and age as factors of GC risk. Regarding prognosis, the results reflect the better performance of the prognostic model compared to the diagnostic one. The final prognostic model combining TNM staging, treatment, and the GRS derived from 23 polymorphisms provides a robust performance, exceeding the 0.7 threshold and demonstrating reasonable calibration on the external validation set (C-index value of 0.713). In this case, the model built with Random Survival Forest was chosen due to its advantage in applying explainability techniques [37].

To our knowledge, this is the first study to develop a final decision support tool for both the diagnosis and prognosis of GC. GastricAITool is based on a multicentre dataset, representing one of the most extensive series (643 HC and 603 GC patients) reported in Caucasian population in which the cohort of GC patients was followed for a long period of time (median: 11.21 months; range: 0.03–120 months). The dataset includes a wide variety of information concerning clinical–demographic, tumoral, environmental, and genetic information. GastricAITool also addresses some limitations in previous studies primarily related to information, usability, generalisation, and model transparency [23,24]. In particular, this is a tool that comprehensively covers most relevant factors influencing GC risk and prognosis. Moreover, several algorithms, both traditional and ML and DL, were explored and validated internally and externally, ensuring a more effective generalisation. Additionally, XAI techniques were employed to provide transparency and interpretability of the final models. These characteristics, along with an intuitive and user-friendly design, mean that GastricAITool holds great promise as a tool in GC decision-making. However, some limitations should also be considered. As mentioned earlier, GC patients and controls were matched by sex and age in our dataset, and therefore their relevance in the prediction of GC risk could not be assessed. To address this aspect in future works, the creation of models considering sex- and age-unmatched datasets is suggested. This approach is expected to lead to a substantial improvement in the predictive ability of the diagnostic model since age and sex are well-documented risk factors for GC development [54]. Additional lifestyle factors related to GC risk such as alcohol consumption or dietary habits (high intake of salt and low intake of fresh fruit and vegetables) were not investigating in our study. The omission of these elements is attributed to challenges in collecting such information that may result in misclassifications and bias. Our study comprises 603 unrelated GC patients recruited at hospitals from May 2003 to August 2012. Throughout this recruitment period, there were changes in treatment practices, mainly related to chemotherapy and radiotherapy guidelines. In our study, we did not assess the possible benefit of neoadjuvant/adjuvant chemotherapy and radiotherapy on the prognosis of GC patients since their schedules varied considerably among the participating hospitals. Therefore, only general information related to the type of treatment received by the patient (surgery, chemo- and radiotherapy) was considered for evaluation in the algorithm. Likewise, data concerning anti-HER2, anti-angiogenic therapies (anti-VEGF), and immune checkpoint blockade (anti-PD-1) were not available at the time of recruitment for most patients. Finally, only common genetic variants were analysed and selected for the construction of the GRS in our diagnostic and prognostic models. Furthermore, future studies considering rare and relevant copy number variants are warranted in order to evaluate their effect on GC predictive models.

Among the limitations of the study, the ability to generalise models to other populations deserves special consideration. Despite following a thorough methodology to build diagnostic and prognostic models for GC, it is important to highlight that these models were trained on a dataset specific to a Spanish population, which has its particular characteristics. Consequently, the validity of the models and the conclusions of this study are determined within this context. It is well known that characteristics between populations vary in terms of geographic location, ethnicity, lifestyle factors, healthcare systems, or genetic factors, among others. Therefore, it is important to note that, before being used in other populations, the models should be validated in those different contexts to ensure an effective generalization.

In future research, we consider exploring the feasibility of implementing a self-learning algorithm (SLA) that, once deployed, can continuously be optimised by training it on new data over time. However, each adjustment or update to the algorithm should undergo a comprehensive validation process to ensure that it maintains its accuracy and clinical acceptability. Furthermore, implementing SLAs must align with the specific legal regulations of each jurisdiction, as regulations on the use of AI technologies in medicine can vary significantly.

In conclusion, this is the first study to develop a final decision support tool for both the diagnosis and prognosis of GC that integrates demographic, environmental, clinical, tumour, and genetic information. The result is an intuitive tool that allows clinicians to input patient data and obtain an assessment of cancer risk and prognosis, accompanied by explanatory graphs of the results provided by the model. As a future line of work, the inclusion of other data related to medical images, clinical information, immunotherapy, and new susceptibility loci is expected to increase both diagnosis and prognosis accuracy. In the near future, GastricAITool might contribute to enhancing the healthcare system, facilitating early cancer detection, and advocating for more effective patient monitoring.

## Figures and Tables

**Table 1:** Clinical and demographic characteristics of the study population.

Characteristics		Controls n = 533	GC Patients n = 490	*p*-Value
**Age** (years)	Mean (SD)	69.96 (12.61)	70.6 (12.58)	0.32
	Median (IQR)	72 (63–79)	73 (64–80)	
**Sex**	Female	171 (32.08%)	154 (31.43%)	0.875
**Smoking**	Never/Former	452 (84.8%)	382 (77.96%)	**0.006**
	Current smoker	81 (15.2%)	108 (22.04%)	
***H. pylori* infection**	Positive	325 (60.98%)	349 (71.22%)	**0.001**
**Family history of GC**	Positive	33 (6.19%)	72 (14.69%)	**<0.001**

n: number of individuals. *p*-values < 0.05 are highlighted in bold.

**Table 2:** Genetic variants selected for the construction of the diagnostic model.

CarrierIlra2 *	rs1060826	rs10759932	rs17655	rs20417	rs2074522	rs2228000
rs2345060	rs4072037	rs4150416	rs4986764	rs569143	rs5788	rs6679677
rs909253	rs9894946					

* CarrierIlra2 refers to a variable number of an 86-base pair tandem repeat polymorphism (VNTR) in intron 2 of the *IL1RN* gene. Individuals were classified according to the carriage of allele 2 of the *ILIRN* gene polymorphism. rs: SNP identification according to the NCBI database.

**Table 3:** Overall survival analysis at different time intervals according to clinicopathological.

Variables	*p*-Value (1.5 Years)	*p*-Value (3 Years)	*p*-Value (5 Years)	*p*-Value (10 Years)
***H. Pylori* Infection**	0.442	0.728	0.735	0.71
**Sex**	0.21	0.112	0.067	0.069
**Age > 50 years**	**0.027**	0.142	0.144	0.088
**Smoking (current)**	0.511	0.858	0.528	0.776
**Family History of GC**	0.572	0.348	0.262	0.169
**Charlson index ≥ 3**	0.742	0.633	0.373	0.265
**Cardial Tumour location**	**0.011**	**0.002**	**0.001**	**0.001**
**Lauren’s Histological type**	0.215	0.362	0.608	0.896
**TNM staging**	**<0.001**	**<0.001**	**<0.001**	**<0.001**
**Metastasis at Diagnosis**	**<0.001**	**<0.001**	**<0.001**	**<0.001**
**T1–T2 vs. T3–T4**	**<0.001**	**<0.001**	**<0.001**	**<0.001**
**N0 vs. N1–N2–N3**	**<0.001**	**<0.001**	**<0.001**	**<0.001**
**Chemotherapy**	**<0.001**	**<0.001**	**<0.001**	**<0.001**
**Radiotherapy**	**<0.001**	**<0.001**	**<0.001**	**<0.001**
**Surgery**	**<0.001**	**<0.001**	**<0.001**	**<0.001**

*p*-values < 0.05 are highlighted in bold.

**Table 4:** Genetic variants selected for the construction of the prognostic model.

CarrierIlra2 *	rs1052133	rs11086565	rs12711521	rs13181	rs144848	rs1799796
rs1800470	rs1898830	rs2074522	rs207906	rs26779	rs2738120	rs2738169
rs293794	rs3088074	rs4072037	rs4234259	rs4986790	rs4987876	rs6151662
rs7744	rs7797466	rs7932766	rs8305	rs9841504		

* CarrierIlra2 refers to a variable number of an 86-base pair tandem repeat polymorphism (VNTR) in intron 2 of the *IL1RN* gene. Individuals were classified according to the carriage of allele 2 of the *ILIRN* gene polymorphism. rs: SNP identification according to the NCBI database.

**Table 5:** Association between unweighted GRS and GC risk.

GRS Values	OR	Lower CI	Upper CI	*p*-Value
**≤15**	**Ref**			
**16**	1.40	0.33	6.48	0.657
**17**	1.03	0.29	4.25	0.966
**18**	1.47	0.46	5.65	0.537
**19**	2.01	0.67	7.46	0.243
**20**	2.52	0.86	9.17	0.117
**21**	3.24	1.11	11.83	**0.046**
**22**	4.59	1.56	16.81	**0.01**
**23**	5.21	1.75	19.28	**0.006**
**24**	5.87	1.94	21.91	**0.003**
**25**	5.69	1.71	22.92	**0.007**
**≥ 26**	12.25	3.17	58.04	**0.001**
**Continuous value**	1.25	1.19	1.33	**<0.001**

*p*-values < 0.05 are highlighted in bold.

**Table 6:** Association between weighted GRS and GC risk.

GRS Values	OR	Lower CI	Upper CI	*p*-Value
**1**	**Ref**			
**2**	1.99	1.38	2.88	<0.001
**3**	3.62	2.51	5.25	<0.001
**4**	4.03	2.79	5.86	<0.001

**Table 7:** Association between unweighted GRS and OS time in GC patients.

GRS Values	HR	Lower CI	Upper CI	*p*-Value
**≤15**	**Ref**			
**16**	0.84	0.45	1.50	0.566
**17**	1.33	0.81	2.20	0.258
**18**	1.22	0.74	2.00	0.438
**19**	1.12	0.69	1.80	**0.012**
**20**	1.79	1.13	2.80	<**0.001**
**21**	2.27	1.41	3.60	<**0.001**
**22**	2.50	1.55	4.00	<**0.001**
**23**	3.72	2.18	6.30	<**0.001**
**24**	7.04	3.80	13.00	<**0.001**
**25**	7.96	3.79	16.70	<**0.001**
**≥26**	10.07	5.24	19.40	<**0.001**
**Continuous value**	1.20	1.20	1.30	<**0.001**

*p*-values < 0.05 are highlighted in bold.

**Table 8:** Association between weighted GRS and OS time in GC patients.

GRS Values	HR	Lower CI	Upper CI	*p*-Value
**1**	**Ref**			
**2**	1.40	1.10	1.90	0.018
**3**	2.20	1.70	3.00	<0.001
**4**	4.00	3.00	5.40	<0.001
**Continuous value**	2.70	2.30	3.20	<0.001

**Table 9:** Diagnosis model comparison. Different combinations of input variables. 5k-fold.

Models	AUC Mean (SD)
(1) Clinical–demographic model	0.606 (0.034)
(2) Univariate model: Unweighted GRS	0.647 (0.034)
(3) Univariate model: Weighted GRS	0.655 (0.033)
(4) Clinical–demographic and unweighted GRS	0.678 (0.036)
**(5) Clinical–demographic and weighted GRS**	**0.682 (0.036)**
(6) Model 4 with interactions with unweighted GRS	0.674 (0.035)
(7) Model 5 with interactions with weighted GRS	0.678 (0.035)
(8) Clinical–demographic and SNPs	0.655 (0.030)

(1) Clinical–demographic: age + sex + smoking + *H. pylori* infection + family history of GC. (2) Unweighted GRS. (3) Weighted GRS. (4) age + sex + smoking + *H. pylori* infection + family history of GC + unweighted GRS. (5) age + sex + smoking + *H. pylori* infection + family history of GC + weighted GRS. (6) age + sex + smoking + *H. pylori* infection + family history of GC + unweighted GRS + interactions with unweighted GRS. (7) age + sex + smoking + *H. pylori* infection + family history of GC + weighted GRS + interactions with weighted GRS. (8) age + sex + smoking + *H. pylori* infection + family history of GC + SNPs. Bold text highlights the model with the highest discrimination capacity.

**Table 10:** Prognosis model’s comparison. Different combinations of input variables. 5k-fold.

Models	AUC Mean (SD)
(1) Clinical–demographic model	0.586 (0.032)
(2) TNM model	0.698 (0.019)
(3) Treatments model	0.623 (0.012)
(4) Univariate model: Weighted GRS	0.664 (0.033)
(5) Non-genetic variable model	0.730 (0.023)
**(6) Total model (weighted GRS + non-genetic variables)**	**0.761 (0.037)**
(7) Total model (unweighted GRS + non-genetic variables)	0.674 (0.021)

(1) Clinical–demographic model: age + sex + tumour location. (2) TNM model: global TNM staging + metastasis. (3) Treatment model: chemotherapy + radiotherapy + surgery. (4) Weighted GRS. (5) Non-genetic model: age + sex + tumour location + global TNM staging + metastasis + chemotherapy + radiotherapy + surgery. (6) weighted GRS + age + sex + tumour location + global TNM staging + metastasis + chemotherapy + radiotherapy + surgery. (7) unweighted GRS + age + sex + tumour location + global TNM staging + metastasis + chemotherapy + radiotherapy + surgery. Bold text highlights the model with the highest performance capacity.

**Table 11:** Model performance. 5k-fold.

Diagnosis		Prognosis	
Models	AUC Mean (SD)	Models	C-Index Mean (SD)
LR	0.679 (0.043)	Cox regression	0.757 (0.011)
Lasso LR	0.679 (0.043)	Lasso Cox	0.758 (0.012)
Ridge LR	0.680 (0.044)	Ridge Cox	0.758 (0.013)
RF	0.670 (0.034)	**RSF**	**0.769 (0.016)**
SVM	0.680 (0.040)	SSVM	0.768 (0.007)
**XGBoost**	**0.684 (0.043)**	Survival XGBoost	0.727 (0.022)
MLP (2) *	0.672 (0.043)	**DeepCox**	**0.773 (0.016)**
MLP (3) +	0.678 (0.044)		

* Two hidden layers. + Three hidden layers. Bold text highlights models with the highest discrimination capacity.

## Data Availability

The genotyping data supporting the current study remain confidential because are currently under study, but they are available from the corresponding author on request. GastricAITool tool is licensed by Safe Creative, register number 2307204867360. The access to the tool is free and available under request (vrodrigalvarez@ita.es).

## Supplementary Materials

The following supporting information can be downloaded at: https://www.mdpi.com/article/10.3390/biomedicines12092162/s1, Table S1: Characteristics of candidate SNPs analysed in this study; Table S2: Search space of the hyperparameters explored for each algorithm. Diagnosis models; Table S3: Search space of the hyperparameters explored for each algorithm. Prognosis models.
