# Supplementary material for: GastricAITool: A Clinical Decision Support Tool for the Diagnosis and Prognosis of Gastric Cancer
Source: Biomedicines. 2024 Sep 23;12(9):2162. doi: 10.3390/biomedicines12092162 (PMC11429470; doi:10.3390/biomedicines12092162)
Supplement: Supplementary file 1 [file biomedicines-12-02162-s001.zip › biomedicines-3178350-supplementary.pdf]

Supplementary Material

Table S1: Characteristics of candidate SNPs analysed in the study.

SNP[a]	Gene	Chr	Position[b]	SNP Type	Alleles[c]	MAF	HWE[d]
rs12711521	*MASP2*	1	11030859	D371Y	A/C	0.21	0.733
rs1801133	*MTHFR*	1	11796321	A263V	G/A	0.34	0.587
rs3219489	*MUTYH*	1	45331833	Q324H	G/C	0.27	0.542
rs1048771	*RAD54L*	1	46278228	A730A	C/T	0.12	0.412
rs6679677	*PHTF1*	1	113761186	Upstream	C/A	0.09	0.231
rs4072037	*MUC1*	1	155192276	T22T	T/C	0.49	0.493
rs1772408	*IFI16*	1	159035859	Intron	G/A	0.16	0.385
rs689469	*PTGS2*	1	186672065	3′ UTR	G/A	0.03	0.372
rs4648298	*PTGS2*	1	186672550	3′ UTR	A/G	0.03	0.389
rs5275	*PTGS2*	1	186673926	3′ UTR	T/C	0.31	0.326
rs5277	*PTGS2*	1	186679065	V102V	G/C	0.19	0.225
rs20417	*PTGS2*	1	186681189	Upstream	G/C	0.21	0.423
rs689466	*PTGS2*	1	186681619	Upstream	A/G	0.19	0.178
rs1800872	*IL10*	1	206773062	Upstream	C/A	0.24	0.917
rs1800896	*IL10*	1	206773552	Upstream	A/G	0.42	0.311
rs5744174	*TLR5*	1	223111186	F616L	A/G	0.37	0.571
rs2072493	*TLR5*	1	223111257	N592S	T/C	0.17	0.687
rs5744168	*TLR5*	1	223111858	R392*	C/T	0.04	0.636
rs1136410	*PARP1*	1	226367601	V762A	T/C	0.13	0.263
rs735943	*EXO1*	1	241866849	H354R	C/T	0.47	0.086
rs4149963	*EXO1*	1	241872080	T439M	C/T	0.08	0.438
rs1047840	*EXO1*	1	241878999	E589K	G/A	0.39	0.932
rs1776148	*EXO1*	1	241879243	E670G	G/A	0.39	0.863
rs9350	*EXO1*	1	241885372	P757L	C/T	0.15	0.215
rs1863332	*MSH2*	2	47402759	Upstream	A/C	0.08	0.032
rs1981928	*MSH2*	2	47445336	Intronic	T/A	0.27	1.000
rs2303428	*MSH2*	2	47476361	Intronic	T/C	0.10	0.153
rs3136228	*MSH6*	2	47782677	Upstream	T/G	0.35	0.717
rs2348244	*MSH6*	2	47792346	Intronic	T/C	0.13	1.000
rs1800935	*MSH6*	2	47795976	D180D	T/C	0.29	0.682
rs2020911	*MSH6*	2	47803699	Intronic	A/T	0.35	0.533
rs1143634	*IL1B*	2	112832813	F74F	C/T	0.21	0.733
rs16944	*IL1B*	2	112837290	Upstream	C/T	0.34	0.353
rs4150474	*ERCC3*	2	127275751	Intronic	T/G	0.24	0.199
rs4150441	*ERCC3*	2	127283339	Intronic	A/G	0.41	0.733
rs4150416	*ERCC3*	2	127288972	Intronic	T/G	0.31	0.457
rs4664308	*PLA2R1*	2	160060986	Downstream	A/G	0.34	0.866
rs2111485	*IFIH1*	2	162254026	Intergenic	G/A	0.39	0.936
rs1990760	*IFIH1*	2	162267541	A946T	T/C	0.40	0.097
rs207906	*XRCC5*	2	216148178	T524T	G/A	0.12	0.351
rs1051677	*XRCC5*	2	216205525	3′ UTR	T/C	0.09	1.000

rs1051685	*XRCC5*	2	216205653	3′ UTR	A/G	0.10	0.062
rs2440	*XRCC5*	2	216206043	3′ UTR	C/T	0.45	0.804
rs10210302	*ATG16L1*	2	233250193	Upstream	T/C	0.48	0.594
rs12994997	*ATG16L1*	2	233264857	Intron	A/G	0.47	0.594
rs3828309	*ATG16L1*	2	233271764	Intron	G/A	0.48	0.543
rs2241880	*ATG16L1*	2	234183368	T300A	G/A	0.47	0.594
rs1052133	*OGG1*	3	9757089	S326C	C/G	0.21	0.632
rs293794	*OGG1*	3	9761943	Intronic	T/C	0.17	0.670
rs2228001	*XPC*	3	14145949	K939Q	A/C	0.39	0.865
rs2228000	*XPC*	3	14158387	A499V	C/T	0.31	0.403
rs1800734	*MLH1*	3	36993455	5′ UTR	G/A	0.24	0.267
rs1540354	*MLH1*	3	37002998	Intronic	T/A	0.12	0.124
rs4234259	*MLH1*	3	37007142	Intronic	A/G	0.49	0.101
rs1799977	*MLH1*	3	37012077	I219V	A/G	0.33	0.360
rs2286940	*MLH1*	3	37028615	Intronic	C/T	0.49	0.120
rs9876116	*MLH1*	3	37042249	Intronic	A/G	0.48	0.087
rs2239621	*ACAA1*	3	38132242	Intron	C/T	0.28	0.349
rs7744	*MyD88*	3	38142530	3′ UTR	A/G	0.14	0.746
rs6853	*MyD88*	3	38142879	3′ UTR	A/G	0.13	1.000
rs1050450	*GPX1*	3	49357401	3′ UTR	C/T	0.33	0.144
rs352140	*TLR9*	3	52222681	P545P	A/G	0.47	0.224
rs5743836	*TLR9*	3	52226766	Upstream	T/C	0.17	0.426
rs187084	*TLR9*	3	52227015	Upstream	A/G	0.40	0.204
rs9841504	*ZBTB20*	3	114643917	Intron	C/G	0.07	0.575
rs1802904	*ATR*	3	142449489	Q2625Q	A/G	0.13	0.264
rs4129009	*TLR10*	4	38773268	I775V	A/G	0.27	0.772
rs11466657	*TLR10*	4	38774173	I473T	A/G	0.07	0.568
rs10004195	*TLR10*	4	38783103	Upstream	T/A	0.35	0.114
rs4833095	*TLR1*	4	38798089	N248S	T/C	0.39	0.577
rs5743551	*TLR1*	4	38806033	Upstream	T/C	0.39	0.749
rs4833103	*TLR1*	4	38813881	Upstream	C/A	0.40	0.478
rs5743810	*TRL6*	4	38828729	S249P	G/A	0.35	0.501
rs4073	*IL8*	4	73740307	Upstream	T/A	0.46	0.879
rs7143468	*TRAF3*	4	102771441	Not reported	G/A	0.33	0.085
rs1898830	*TLR2*	4	153687301	Intron	A/G	0.31	0.788
rs3804099	*TLR2*	4	153703504	N199N	T/C	0.47	0.647
rs3804100	*TLR2*	4	153704257	S450S	T/C	0.08	1.000
rs5743708	*TLR2*	4	153705165	R753Q	G/A	0.01	0.064
rs11721827	*TLR3*	4	186069983	Intron	A/C	0.10	10000
rs3775292	*TLR3*	4	186081871	Intron	G/C	0.22	0.221
rs3775291	*TLR3*	4	186082920	L412F	C/T	0.37	0.141
rs6872282	*PTGER4*	5	40703988	Intronic	C/T	0.24	0.823
rs10074991	*PRKAA1*	5	40790449	Intron	G/A	0.33	0.030
rs13361707	*PRKAA1*	5	40791782	Intron	C/T	0.32	0.069
rs1650697	*MSH3*	5	80654962	V79T	C/T	0.25	0.458
rs6151662	*MSH3*	5	80677451	Intronic	G/A	0.05	0.615
rs26779	*MSH3*	5	80763384	Intronic	G/A	0.39	0.398

rs10079641	*MSH3*	5	80798145	Intronic	C/G	0.10	0.381
rs26279	*MSH3*	5	80873118	A1045T	A/G	0.32	0.110
rs6869366	*XRCC4*	5	83075927	Upstream	T/G	0.06	0.659
rs2075685	*XRCC4*	5	83076846	Upstream	G/T	0.44	1.000
rs1478485	*XRCC4*	5	83084708	Intronic	C/T	0.41	0.865
rs13180316	*XRCC4*	5	83163634	Intronic	G/A	0.27	1.000
rs963248	*XRCC4*	5	83238075	Intronic	A/G	0.16	1.000
rs2252775	*RAD50*	5	132582752	Intronic	A/C	0.21	0.062
rs2243250	*IL4*	5	132673462	Upstream	C/T	0.18	0.514
rs2569190	*CD14*	5	140633331	Upstream	G/A	0.48	0.116
rs2569192	*CD14*	5	140635623	Upstream	G/C	0.28	0.349
rs1000113	*IRGM*	5	150860514	Downstream	C/T	0.15	0.010
rs3212227	*IL12p40*	5	159315942	3′ UTR	A/C	0.22	0.502
rs909253	*LTA*	6	31540313	Intron	A/G	0.26	0.329
rs746868	*LTA*	6	31572652	Intron	C/G	0.42	0.212
rs1800629	*TNFA*	6	31575254	Upstream	G/A	0.12	0.152
rs361525	*TNFA*	6	31575324	Upstream	G/A	0.10	0.507
rs2294693	*UNC5CL*	6	41037763	Intron	T/C	0.17	0.786
rs6458238	*PGC*	6	41749967	Not reported	G/A	0.08	0.204
rs4880	*SOD2*	6	159692840	V16A	T/C	0.47	0.128
rs2228006	*PMS2*	7	5987144	K541E	G/A	0.16	0.053
rs2345060	*PMS2*	7	5999498	Intronic	A/G	0.24	1.000
rs7797466	*PMS2*	7	6007152	Intronic	G/A	0.16	0.056
rs1800795	*IL6*	7	22727026	Upstream	G/C	0.34	0.091
rs2075821	*CARD4/NOD1*	7	30451695	A574A	C/T	0.25	0.229
rs2075820	*CARD4/NOD1*	7	30452621	E266K	C/T	0.25	0.220
rs1799983	*NOS3*	7	150999023	D298E	G/T	0.32	0.471
rs3218536	*XRCC2*	7	152648922	R188H	G/A	0.10	0.253
rs2040639	*XRCC2*	7	152678103	Upstream	G/A	0.45	0.566
rs2741112	*DEFB1*	8	6867995	Upstream	C/G	0.43	0.816
rs2978858	*DEFB1*	8	6883094	5′ UTR	G/C	0.10	0.665
rs2738169	*DEFB1*	8	6885036	5′ UTR	G/A	0.12	0.074
rs13275170	*DEFA6*	8	6922722	Upstream	C/T	0.49	0.762
rs2738120	*DEFA6*	8	6925114	Intron	C/G	0.34	0.735
rs1800389	*WRN*	8	31067041	C171C	T/C	0.26	0.214
rs1346044	*WRN*	8	31167138	C1367R	T/C	0.22	0.563
rs2953993	*POLB*	8	42342820	Intronic	C/T	0.07	0.167
rs2272615	*POLB*	8	42361530	Downstream	A/G	0.12	0.449
rs3136797	*POLB*	8	42369287	P242R	C/G	0.05	0.725
rs1805794	*NBS1*	8	89978251	E185Q	C/G	0.30	0.138
rs2294008	*PSCA*	8	142680513	M1W	C/T	0.45	0.321
rs10813831	*DDX58*	9	32526148	Arg7Cys	G/A	0.29	0.515
rs1800975	*XPA*	9	97697296	5′ UTR	G/A	0.31	0.637
rs10759932	*TLR4*	9	117702866	Upstream	T/C	0.15	1.000
rs2149356	*TLR4*	9	117711921	Intron	G/T	0.32	0.794
rs4986790	*TLR4*	9	117713024	D299G	A/G	0.06	0.505
rs4986791	*TLR4*	9	117713324	T399I	C/T	0.07	0.351

rs11536889	*TLR4*	9	117715853	3′ UTR	G/C	0.15	0.2255
rs1330344	*PTGS1*	9	122369409	Upstream	A/G	0.21	0.257
rs3842787	*PTGS1*	9	122371228	P17L	C/T	0.05	1.000
rs5788	*PTGS1*	9	122381513	G213G	C/A	0.17	0.072
rs10781499	*CARD9*	9	136371953	P42P	G/A	0.38	0.226
rs10781500	*CARD9*	9	136374886	Upstream	C/T	0.37	0.292
rs2437258	*MRC1*	10	17891822	Intron	C/T	0.25	0.221
rs1926736	*MRC1*	10	17931711	G396S	G/A	0.41	0.568
rs4253160	*ERCC6*	10	49485920	Intronic	A/T	0.45	0.461
rs2228524	*ERCC6*	10	49532830	L45L	G/C	0.26	0.437
rs3793784	*ERCC6*	10	49539493	5′ UTR	C/G	0.42	0.319
rs2274223	*PLCE1*	10	94306584	H1927R	A/G	0.35	0.504
rs12917	*MGMT*	10	129708019	L84F	C/T	0.14	0.865
rs2308321	*MGMT*	10	129766800	I174V	A/G	0.09	0.014
rs3793964	*TOLLIP*	11	1280752	Intron	C/T	0.44	0.590
rs3750920	*TOLLIP*	11	1288726	P139P	C/T	0.40	0.578
rs5743899	*TOLLIP*	11	1302334	Intron	T/C	0.22	0.230
rs5743867	*TOLLIP*	11	1307121	Intron	A/G	0.12	1.000
rs1001179	*CAT*	11	34438684	Upstream	C/T	0.22	0.913
rs331457	*TRAF6*	11	36502062	Intron	C/T	0.12	0.726
rs4755453	*TRAF6*	11	36509094	Intron	G/C	0.15	0.289
rs331455	*RAG1*	11	36518999	Intron	T/C	0.26	0.622
rs2434470	*ALKBH3*	11	43919052	E228D	C/G	0.23	0.911
rs174538	*FEN1*	11	61792609	Upstream	G/A	0.30	0.558
rs1695	*GSTP1*	11	67585218	I105V	A/G	0.34	0.497
rs1138272	*GSTP1*	11	67586108	A114V	C/T	0.05	0.400
rs601341	*MRE11A*	11	94434611	Intronic	G/A	0.43	0.408
rs569143	*MRE11A*	11	94455221	Intronic	C/G	0.49	0.807
rs4987876	*ATM*	11	108221910	Intronic	G/T	0.09	0.085
rs664677	*ATM*	11	108272455	Intron	T/C	0.45	0.801
rs1800889	*ATM*	11	108292760	P1526P	C/T	0.05	0.091
rs1801516	*ATM*	11	108304735	D1853N	G/A	0.14	0.602
rs664143	*ATM*	11	108354934	Intronic	C/T	0.37	0.932
rs8177374	*TIRAP*	11	126292948	S180L	C/T	0.18	0.432
rs7932766	*TIRAP*	11	126292967	A186A	C/T	0.21	0.415
rs11226	*RAD52*	12	912647	3′ UTR	C/T	0.43	0.864
rs6413436	*RAD52*	12	913513	Intronic	T/C	0.34	0.926
rs1461567	*IRAK4*	12	43770886	Intron	G/A	0.27	0.034
rs3794262	*IRAK4*	12	43771627	Intron	T/A	0.14	0.625
rs4251545	*IRAK4*	12	43786492	A428T	G/A	0.12	0.726
rs1059262	*ALKBH2*	12	109087930	3′ UTR	T/G	0.18	0.789
rs33962311	*USP30*	12	109088384	Downstream	C/T	0.05	0.597
rs11068458	*NOS1*	12	117364937	Upstream	A/G	0.05	0.705
rs5744934	*POLE*	12	132643940	S1396N	A/G	0.17	1.000
rs144848	*BRCA2*	13	32332592	N372H	T/G	0.29	0.154
rs1801406	*BRCA2*	13	32337751	K1132K	A/G	0.30	0.634
rs1799955	*BRCA2*	13	32355095	S2414S	A/G	0.22	0.484

rs1047768	*ERCC5*	13	102852167	H46H	C/T	0.42	0.452
rs17655	*ERCC5*	13	102875652	D1104H	C/G	0.26	0.540
rs1805386	*LIG4*	13	108209565	D568D	T/C	0.17	0.662
rs1805388	*LIG4*	13	108211243	T9I	C/T	0.14	1000
rs1760944	*APEX1*	14	20454990	Upstream	C/A	0.38	0.364
rs1130409	*APEX1*	14	20456995	D148E	T/G	0.48	1.000
rs175080	*MLH3*	14	75047125	L844P	G/A	0.44	0.280
rs12147254	*TRAF3*	14	102799329	Intron	G/A	0.30	0.072
rs11160707	*TRAF3*	14	102908373	Downstream	G/A	0.01	1.000
rs861539	*XRCC3*	14	103699416	T241M	C/T	0.37	0.603
rs1799796	*XRCC3*	14	103699590	Intronic	A/G	0.23	0.375
rs861531	*XRCC3*	14	103706470	Intronic	G/T	0.39	0.794
rs1799794	*XRCC3*	14	103712930	5´ UTR	A/G	0.25	0.669
rs861528	*XRCC3*	14	103716661	Upstream	C/T	0.24	0.620
rs861528	*XRCC3*	14	103716661	Intronic	G/A	0.25	0.727
rs1801320	*RAD51*	15	40695330	Intronic	G/C	0.08	0.149
rs7182283	*NEIL1*	15	75351418	Intronic	G/T	0.47	0.163
rs176641	*POLG*	15	89346951	Upstream	A/C	0.36	0.589
rs11865121	*CLEC16A*	16	11072831	Downstream	C/A	0.34	0.310
rs12708716	*CLEC16A*	16	11086016	Downstream	A/G	0.38	0.630
rs12924729	*CLEC16A*	16	11093926	Intron	G/A	0.34	0.208
rs998592	*CLEC16A*	16	11105821	Intron	C/T	0.45	0.755
rs3136038	*ERCC4*	16	13919522	Upstream	C/T	0.36	0.262
rs2238463	*ERCC4*	16	13924045	Intronic	C/G	0.38	0.269
rs1800067	*ERCC4*	16	13935176	R415Q	G/A	0.12	0.698
rs1799801	*ERCC4*	16	13948101	S835S	T/C	0.30	0.925
rs2066844	*CARD15/NOD2*	16	50712015	R702W	C/T	0.05	0.690
rs2066845	*CARD15/NOD2*	16	50722629	G908R	G/C	0.015	1.000
rs2066847	*CARD15/NOD2*	16	50729868	fs1007	Del/insC	0.012	1.000
rs9894946	*TP53*	17	7667762	Intronic	G/A	0.15	0.771
rs1614984	*TP53*	17	7668134	Downstream	C/T	0.40	0.727
rs1042522	*TP53*	17	7676154	P72R	G/C	0.25	0.41
rs1060826	*NOS2*	17	27762841	T919T	G/A	0.44	0.038
rs2297518	*NOS2*	17	27769571	S608L	C/T	0.17	0.054
rs2074522	*LIG3*	17	35002629	Intronic	G/A	0.08	0.565
rs17138478	*HNF1B*	17	37713312	Intronic	C/A	0.13	0.834
rs1799966	*BRCA1*	17	43071077	S1613G	A/G	0.34	0.784
rs3737559	*BRCA1*	17	43082287	Intronic	G/A	0.07	1000
rs1060915	*BRCA1*	17	43082453	S1436S	T/C	0.34	0.781
rs16942	*BRCA1*	17	43091983	K1183R	T/C	0.32	0.662
rs16941	*BRCA1*	17	43092418	E1038G	T/C	0.32	0.612
rs799917	*BRCA1*	17	43092919	P871L	C/T	0.36	0.529
rs16940	*BRCA1*	17	43093220	L771L	A/G	0.32	0.568
rs1799950	*BRCA1*	17	43094464	Q356R	T/C	0.07	0.332
rs4986764	*BRIP1*	17	61685986	S919P	C/T	0.38	0.107
rs4968451	*BRIP1*	17	61849946	Intronic	A/C	0.17	0.778
rs2048718	*BRIP1*	17	61863458	Upstream	C/T	0.42	1.000

rs3730668	*POLI*	18	54269477	Upstream	G/T	0.42	0.080
rs8305	*POLI*	18	54294435	A731T	A/G	0.27	0.672
rs8094402	*MBP*	18	76995493	Downstream	A/G	0.38	0.747
rs6510827	*TRIF*	19	4830616	Intron	C/T	0.39	0.204
rs4804800	*CD209*	19	7740242	3′ UTR	A/G	0.12	0.855
rs2287886	*CD209*	19	7747650	Upstream	G/A	0.31	0.052
rs4804803	*CD209*	19	7747847	Upstream	A/G	0.23	0.233
rs735239	*CD209*	19	7748382	Upstream	A/G	0.36	0.100
rs4804805	*CD209*	19	7751660	Not reported	A/G	0.39	0.473
rs6523	*INSL3*	19	17821329	T60P	C/T	0.38	0.377
rs1800471	*TGFB*	19	41352971	R25P	G/C	0.06	1.000
rs1800470	*TGFB*	19	41353016	P10L	T/C	0.42	0.161
rs25487	*XRCC1*	19	43551574	Q399R	G/A	0.37	0.147
rs1799782	*XRCC1*	19	43553422	R194W	G/A	0.06	0.653
rs3213245	*XRCC1*	19	43575535	5′ UTR	T/C	0.39	0.795
rs13181	*ERCC2*	19	45351661	K751Q	T/G	0.34	0.200
rs1799793	*ERCC2*	19	45364001	D312N	G/A	0.32	0.445
rs238406	*ERCC2*	19	45365051	R156R	G/T	0.50	0.121
rs1618536	*ERCC2*	19	45368348	Intronic	G/A	0.48	0.031
rs3212986	*ERCC1*	19	45409478	Q504K	G/T	0.26	0.524
rs3212961	*ERCC1*	19	45419065	Intronic	C/A	0.12	0.550
rs3212948	*ERCC1*	19	45421104	Intron 3	C/G	0.37	0.136
rs20580	*LIG1*	19	48151296	A170A	C/A	0.49	0.287
rs6054706	*DEFB126*	20	149223	Not reported	T/C	0.41	0.433
rs13042395	*SLC52A3*	20	773867	Intron	C/T	0.08	0.603
rs3626	*PCNA*	20	5115125	3′ UTR	G/C	0.13	0.593
rs1980499	*BMP2*	20	6765851	Upstream	T/C	0.47	0.196
rs5743507	*BPI*	20	38310650	V182V	G/C	0.12	1.000
rs4358188	*BPI*	20	38318446	G216K	G/A	0.44	0.694
rs2232582	*LBP*	20	38350862	P97P	T/C	0.14	0.267
rs11086565	*LBP*	20	38359276	Intron	A/G	0.04	0.139
rs2232618	*LBP*	20	38373117	P436L	T/C	0.09	0.814
rs3088074	*ATRX*	X	77682471	E929*	G/C	0.30	0.387

Chr: Chromosome. MAF: Minor allele frequency. [a]SNP identification according to the NCBI data base. [b]Chromosome position according to the Genome Reference Consortium Human Build 38.p14 (GRCh38.p14). [c]Major/minor alleles. [d]Hardy–Weinberg equilibrium p values in control population.

Table S2: Search space of the hyperparameters explored for each algorithm. Diagnosis models.

Model	Hyperparameter search space	Best model (5k-fold)
Logistic Lasso regression	C = [1e-3, 10]	5.743
	tol = [1e-6, 1e-1]	3.35E-05
Logistic Ridge regression	C = [1e-3, 10]	0.2203
	tol = [10e-6, 1e-1]	0.0177
Random Forest	n_estimators = [5, 1500]	80
	max_depth = [1, 500]	387
	nin_sample_split = [2, 50]	40
	min_sample_leaf = [1, 25]	25
SVM	kernel = linear, rbf, sigmoid	linear
	C = [1e-3, 10]	9.6828
	gamma = [1e-5, 10]	1.8814
	tol = [1e-6, 1e-1]	0.00014
XGBoost	n_estimators = [5,1500]	1040
	max_depth = [1, 500]	1
	nin_sample_split = [2, 50]	29
	min_sample_leaf = [1, 25]	10
	learning_rate = [1e-5, 1]	0.4686
	lambda = [1e-5, 1]	0.087
	alpha = [1e-5, 1]	0.1411
	gamma = [1e-5, 1]	0.058
	subsample = [0.25, 1]	0.73
	colsample_bytree = [0.25, 1]	0.5167
MLP (two hidden layers)	learning_rate_init = [1e-4, 0.1]	0.085
	neurons = [10, 100]	First hidden layer = 30
		Second hidden layer = 70
	Activation = identity, tanh, relu	identity
MLP (three hidden layers)	learning_rate_init = [1e-4, 0.1]	0.0901
	neurons = [10, 100]	First hidden layer = 10
		Second hidden layer = 80
		Third hidden layer = 30
	Activation = identity, tanh, relu	identity

Table S3: Search space of the hyperparameters explored for each algorithm. Prognosis models.

Model	Hyperparameter search space	Best model (5k-fold)
Cox Lasso regression	penalizer = [1E-8, 1]	0.1336
Cox Ridge regression	penalizer = [1E-8, 1]	0.2106
Random Survival Forest	n_estimators = [5, 1500]	35
	max_depth = [1, 500]	63
	nin_sample_split = [2, 50]	5
	min_sample_leaf = [1, 25]	8
Survival Support Vector Machine	kernel = linear, rbf, sigmoid	linear
	C = [1E-3, 10]	5.080
	gamma = [1E-5, 10]	2.128E-05
	tol = [1E-6, 1E-1]	9.186E-05
Survival XGBoost	n_estimators= [5, 1500]	65
	max_depth = [1, 500]	184
	learning_rate = [1E-5, 1]	0.013
	lambda = [1E-5, 1]	1.29E-05
	alpha = [1E-5, 1]	0.116
	gamma = [1E-5, 1]	0.11
	colsample_bytree = [0.25, 1]	0.594
Multilayer Perceptron (DeepCox)	learning_rate_init = [1E-4, 0.1]	0.02
	neurons = [10, 100]	20
	Activation = sigmoid, tanh, relu	sigmoid
